# Phytosterols: Potential Metabolic Modulators in Neurodegenerative Diseases

**DOI:** 10.3390/ijms222212255

**Published:** 2021-11-12

**Authors:** Niti Sharma, Mario A. Tan, Seong Soo A. An

**Affiliations:** 1 Bionano Research Institute, Gachon University, 1342 Seongnam-daero, Sujeong-gu, Seongnam-si 461-701, Gyeonggi-do, Korea; nitisharma@gachon.ac.kr; 2 Research Center for the Natural and Applied Sciences, College of Science, University of Santo Tomas, Manila 1015, Philippines; matan@ust.edu.ph

**Keywords:** phytosterols, neurodegeneration, Alzheimer’s disease, natural products, blood brain barrier

## Abstract

Phytosterols constitute a class of natural products that are an important component of diet and have vast applications in foods, cosmetics, and herbal medicines. With many and diverse isolated structures in nature, they exhibit a broad range of biological and pharmacological activities. Among over 200 types of phytosterols, stigmasterol and β-sitosterol were ubiquitous in many plant species, exhibiting important aspects of activities related to neurodegenerative diseases. Hence, this mini-review presented an overview of the reported studies on selected phytosterols related to neurodegenerative diseases. It covered the major phytosterols based on biosynthetic considerations, including other phytosterols with significant in vitro and in vivo biological activities.

## 1. Introduction

Neurodegenerative disease (ND) is a group of disorders (Alzheimer’s, Parkinson’s, multiple sclerosis, amyotrophic lateral sclerosis, Huntington’s disease, prion disease, etc.) that result in progressive degeneration of structure or function of the neurons. Neurodegeneration may progress differently with various levels and locations of neuronal circuitry in the brain, ranging from molecular to systemic pathways.

Alzheimer’s disease (AD) is the most common form of dementia, characterized by progressive memory loss, cognitive impairment, and behavioral complications. Pathophysiologically, AD is characterized by excessive amyloid-*beta* (Aβ) peptide aggregation, intracellular neurofibrillary tangles, highly phosphorylated tau protein, deficiency of essential neurotransmitters, and oxidative stress-induced neuronal damage [1,2,3]. To date, the FDA approved drugs have included medicines that may help minimize or alleviate symptoms by regulating chemicals implicated in neurotransmission, such as acetyl cholinesterase inhibitors (donepezil, galantamine, and rivastigmine) and glutamate receptor antagonists (memantine). Aducanumab (marketed as Aduhelm, an anti-amyloid antibody intravenous (IV) infusion therapy), a new drug recently approved by the FDA, may delay clinical degeneration with benefits to both cognitive and motor function in patients with AD. However, all of these treatments suffer from side effects ranging from headache, nausea, confusion, dizziness, falls, and amyloid-related imaging abnormalities (ARIA; including swelling in the brain and micro hemorrhaging/superficial siderosis). This justified the screening of new and safe therapeutic agents from natural sources. In this context, several plants and their bioactive components were reported to inhibit Aβ formation, cholinesterases, tau proteins aggregation, and free radicals [4,5,6,7]. Various phytosterols are anti-inflammatory in action and are known to abrogate production and activation of nitric oxide (NO), tumor necrosis factor-α (TNF-α), cyclooxygenase-2 (COX-2), inducible nitric oxide synthase (iNOS), and phosphorylated extracellular signal-regulated protein kinase (p-ERK) [8]. Hence, phytosterols may possess therapeutic implications in neurodegenerative diseases.

Phytosterol is a collective term for plant sterols and their saturated forms (stanols). They are present in dietary sources such as unrefined plant oils, nuts, seeds, and legumes [9]. Phytosterols can be called ‘plant cholesterol’ due to their structural similarity to cholesterol. An additional methyl group (campesterol) or ethyl group (sitosterol) at the C-24 position or an additional double bond at the C-22 position (brassicasterol or stigmasterol) differentiate them from cholesterol. Depending on the nature of the diet, sitosterol (65%), campesterol (23%), and stigmasterol (10%) belong to the major group among over 200 types of phytosterols [10]. Other important phytosterols such as brassicasterol, avenasterol, and fucosterol, along with the saturated forms of phytosterols (sitostanol, campestanol, and stigmastanol), are present in minor quantities in the diet. The dietary intake of phytosterols varies from 150–450 mg/day [11], but their intestinal absorption is less than 2% (phytosterols) and 0.2% (phytostanols) in comparison to cholesterol, which is nearly 50% [12,13]. As a result of low absorption and rapid biliary elimination, physiological concentrations of phytosterols in the plasma are on the order of 10^−3^ in comparison to cholesterol [14].

Phytosterols have recently gained the attention of researchers due to cholesterol and lipid lowering, anti-atherogenic and immunomodulating properties, especially with a report on the changes in cholesterol metabolism of patients with AD [15,16,17]. Essentially, there is rarely any information in association with their toxicity apart from the indistinct anabolic effect for some [18], and they have already been declared safe (up to 3 g/day) by the Food and Drug Administration (FDA) and the European Union Scientific Committee (EUSC) [19].

Phytosterols have the ability to cross the blood–brain barrier (BBB) and accumulate in the brain [20]. Hence, they might have a significant role in modulating various pathways in the brain linked to neurodegeneration. As the research or review reports on this important aspect were limited, herein, the influences of phytosterols in neurodegenerative diseases were reviewed and discussed. The most prevalent plant sterols, stigmasterol, β-sitosterol, and campesterol [21], were mainly discussed with several minor sterols— brassicasterol, lanosterol, 24(*S*)-saringosterol, 4,4-dimethyl phytosterols, and ergosterol—for their action mechanisms in potentially treating AD.

## 2. Biosynthesis of the Phytosterols in Plants

**Figure 1:**
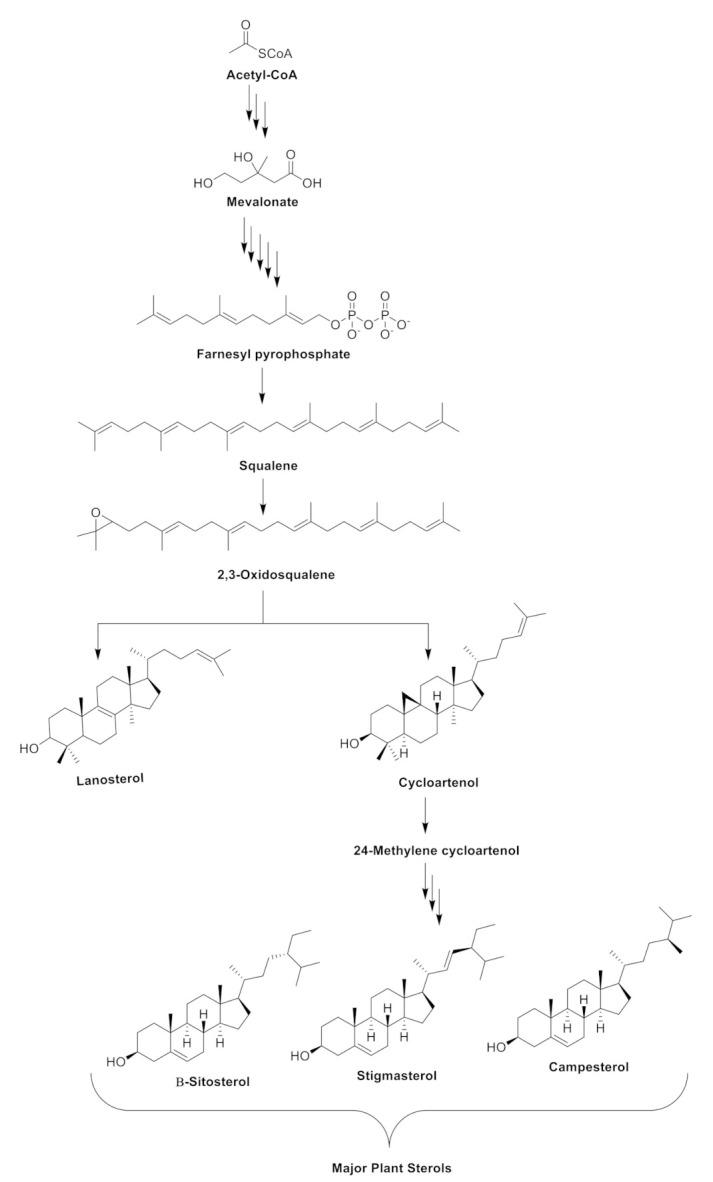
A simplified overview of the biosynthetic pathway of the major phytosterols in plants.

The biosynthesis of phytosterols in plants consists of three major steps. As highlighted in Zhang et al. [22], step one involves the mevalonate or isoprenoid pathway. With acetyl-CoA as the starting point, the process lead to the formation of mevalonate, which serves as an intermediate in the biosynthesis of isoprenoids. Step two is described as the cyclization of squalene and its subsequent conversion to 2,3-oxidosqualene, generating various triterpenes and the phytosterols (lanosterol and cycloartenol). In step three, cycloartenol is converted to 24-methylene cycloartenol by the action of C-24-sterol methyltransferase 1 (SMT1). As shown in Figure 1, the formation of the 24-methylene cycloartenol would facilitate the biosynthesis of the major plant sterols β-sitosterol, stigmasterol, and campesterol.

## 3. Plant Sterols in the Brain

**Figure 2:**
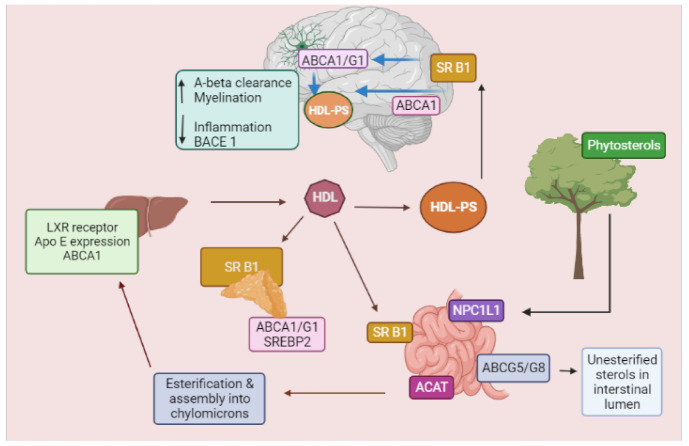
Pathway of dietary phytosterol assimilation in humans. Phytosterols and dietary cholesterol are mainly absorbed in the intestine through NPC1L1 transporter. After absorption, sterols are esterified by ACAT and transported to the liver in the form of chylomicrons. The unesterified sterols are pumped out by ABCG5/ABCG8 transporters. The plasma efflux of sterols is regulated by ABCA1, which is involved in the assembly of HDL-like particles with the assimilated sterols. In liver, phytosterols stimulate LXR receptors regulating ApoE expression required for HDL and LDL assembly and uptake. Stimulated LXR receptors upregulate ABCG5/G8 transporters and augment sterol absorption. The exported HDL-like particles containing phytosterols are taken up by SR-IB receptors expressed on the liver, adrenal, and brain surface. SR-B1 has a crucial role in the flux of phytosterols across the BBB. ABCA1/ABCG1 transporters present on astrocytes and the basolateral side of the cerebral endothelium also assist in the transfer of phytosterols. Inside the brain, PS exert a positive effect on brain function by escalating A-β plaque clearance, increasing re-myelination besides reducing neuro inflammation and BACE 1 activity. Abbreviations: ABC, ATP-binding cassette transporters; A-β, Amyloid-β; ACAT, Acyl-coenzyme A cholesterol acyl transferase; ApoE, Apolipoprotein E; BACE1, β-Secretase 1; HDL, High-density lipoprotein; SR-B1, Scavenger receptor class B type 1; LDL, Low-density lipoprotein; LXR, Liver X receptor; NPC1L1, Niemann-Pick C1 like 1 protein; PS, Phytosterols.

As the exact mechanism through which phytosterols cross the BBB is still not fully understood, it was speculated that high density lipoprotein (HDL)-like particles carrying plant sterols and apolipoprotein E (ApoE) assist this process in brain [18,23,24]. The HDL-like particles are transported through the scavenger receptor class B type 1 (SR-B1) HDL receptors, present on the apical side of the BBB and released into the brain through ATP-binding cassette (ABCA/ABCG1) transporters, on the basolateral side of the BBB and on the astrocytes membrane. Low density and very low density lipoprotein (LDL/VLDL) receptors could transfer these HDL-like particles loaded with plant sterols on the surface of microglia and oligodendrocytes (Figure 2). Even though the brain accounts for 2.1% of the total body weight, it contains nearly 23% of all free cholesterol [25], which is synthesized by the brain itself (in situ), because the cholesterol in the circulation will not cross the BBB. A steady cholesterol turnover plays a critical role in synapse formation, cell–cell interactions, and intracellular signaling [25,26]. The level of cholesterol is maintained in the brain by removal of excess cholesterol out of the BBB in the form of polar 24-(*S*)-OH-cholesterol [27]. On the other hand, the metabolism of phytosterols is entirely different in the brain. Due to the presence of alkyl groups at C-24, they cannot be converted to more polarized derivatives. Therefore, after entering the brain circulation, phytosterols are irretrievably accumulated and incorporated into the cell membranes [28], where they display a positive role in reducing inflammation, Aβ levels, and β-secretase activity [29]. Again, the mechanism is still not confirmed, but is expected to occur through peroxisome proliferator-activated receptors (PPAR) signaling, involving the activation of liver X receptor/retinoid X receptor (LXR/RXR) and the regulation of apolipoprotein E (ApoE) [18]. The sterols from Aloe vera were reported to act through PPAR receptors and increased the expression of fatty acid transporter (FATP1), acyl-CoA- oxidase 1 (ACOX1), and carnitine palmitoyl transferase 1 (CPT1) in a dose-dependent manner, as well as by increasing glutathione and decreasing IL-18 expression [30]. Cholesterol is highly amyloidogenic; however, stigmasterol (having an additional double-bond at C-22/C-23 and an ethyl-group at C-24 in cholesterol skeleton) significantly decreased Aβ levels [31]. Evidence linked raised cholesterol levels to increased Aβ generation in cellular and most animal models of AD [32], and studies have indicated that drugs that inhibit cholesterol synthesis also lower Aβ in these models [33]; however, some other results are contradictory [34]. Studies showed that changes in cholesterol homeostasis, distribution within neurons, and compartmentation affected the processing of APP and Aβ generation [35]. The identification of a variant of the apolipoprotein E (*APOE*) gene as a major genetic risk factor for AD is also consistent with a role for cholesterol in the pathogenesis of AD [36]. The activity of an enzyme responsible for clearing cholesterol from peripheral cells, lecithin cholesterol acyltransferase (LCAT), is significantly decreased in atherosclerosis and AD [37].

**Figure 3:**
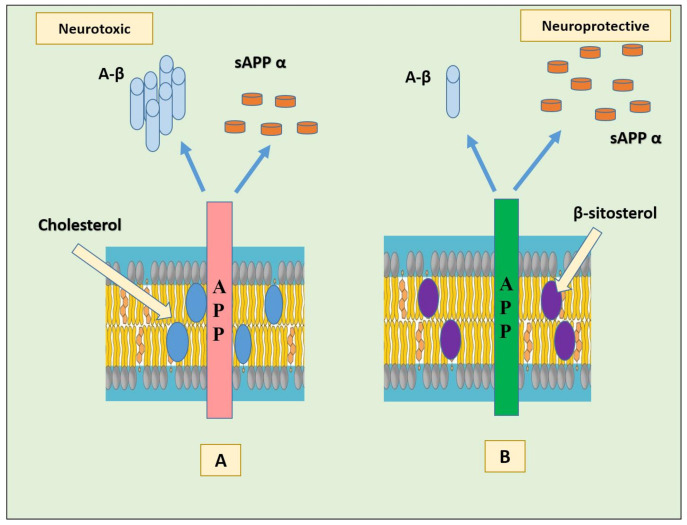
(**A**) Membrane cholesterol favors β-secretase cleavage of APP by direct binding to the C-terminal transmembrane domain of APP, generating neurotoxic Aβ and less sAPP α. (**B**) Substitution of membrane cholesterol with β-sitosterol promotes the re-distribution of APP in non-raft region and non-amyloidogenic processing, generating less Aβ and more of neuroprotective sAPP α. Abbreviations: Aβ: beta amyloid; APP: amyloid precursor protein; sAPP α: soluble alpha-amyloid precursor protein.

In particular, binding of cholesterol to the C-terminal transmembrane domain (C99, also known as the β-CTF) of the amyloid precursor protein (APP) seems to favor the amyloidogenic pathway in cells by endorsing localization of C99 in lipid rafts where γ-secretase and possibly β-secretase are present [38]. The products formed (amyloid-β and the intracellular domain of the APP (AICD)) might possibly down-regulate ApoE-mediated cholesterol uptake and cholesterol biosynthesis. Another possible mechanism by which cholesterol/C99 promote amyloidogenesis is via directly modifying substrate binding, catalysis, or product dissociation by β- or γ-secretase. The activity of these enzymes is much higher in the presence of cholesterol than in its absence [39]. The APP appears as a cellular cholesterol sensor; when membrane cholesterol levels are high, APP binds cholesterol to form a complex and promote the amyloidogenic pathway [40]. In a study using HT22 mouse hippocampal cells, the effect of substituting cholesterol with β- sitosterol was evaluated on APP metabolism. The results showed that the substitution stimulated non-amyloidogenic APP processing possibly by redistribution of APP from lipid rafts toward non-raft regions, without affecting membrane fluidity (Figure 3) [41].

In fact, they significantly reduced brain Aβ levels and β- and γ-secretase activities in vivo, suggesting that a diet enriched in plant sterols might be beneficial for AD. Additionally, the administration of a blend of sitosterol (60%), campesterol (25%), and stigmasterol (15%) has been shown to have an anti-inflammatory effect on central nervous system (CNS) demyelination in an autoimmune encephalomyelitis (EAE) model of multiple sclerosis (MS) [42]. The similar outcomes were expected for AD, as both AD and MS were tightly related in lipid metabolism. Phytosterols are also known to prevent disease progression and to improve motor defects by increasing myelin content by facilitating incorporation of proteolipid protein (PLP) into myelin membranes, improving density of oligodendrocytes and reducing inflammation in animal models [43,44]. RT-PCR results indicated the role of phytosterols in modulating the action of a variety of growth factors from their involvement in the differentiation and survival of oligodendrocyte precursor cells [43]. Phytosterol supplementation elevated the expression levels of fibroblast growth factor 1 (FGF1) and Sonic hedgehog (SHH), without affecting insulin-like growth factor 1 (IGF1), epidermal growth factor (EGF), and ciliary neurotrophic factor (CNTF).

## 4. Neuroprotective Effects of the Selected Phytosterols

**Figure 4:**
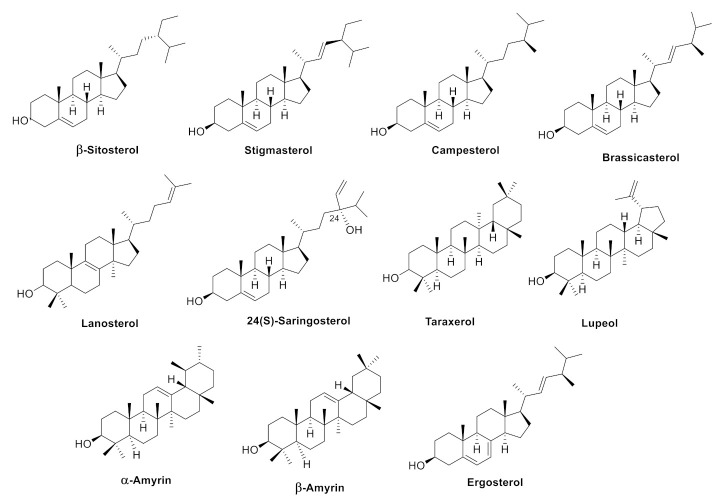
Structure of the phytosterols with potential activities against neurodegenerative diseases.

**Figure 5:**
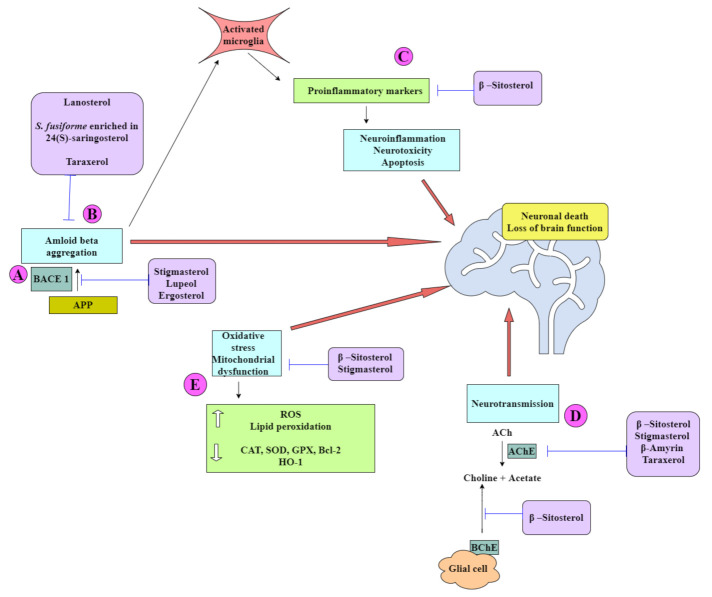
Phytosterols exert a multitarget approach to ameliorate symptoms of AD. (**A**) Some prevent amyloid-*beta* aggregation by inhibiting cleavage of the amyloid precursor protein (APP) by β-secretase (BACE-I). This causes a shift in the non-amyloidogenic pathway and reduces the levels of Aβ produced. (**B**) Aβ can self-aggregate to form oligomers and eventually amyloid plaques. Some phytosterols are able to inhibit the formation of amyloid plaques by binding to Aβ, inhibiting aggregation, and thereby promoting the formation of nontoxic oligomers. Toxic Aβ monomers and oligomers have been shown to induce microglial activation and proliferation. Activated microglia secrete pro-inflammatory cytokines such as IL-1β and IL-6. (**C**) Some phytosterols have been shown to reduce the levels of these cytokines. Some phytosterols reduce oxidative stress by increasing the levels of antioxidant enzymes and reducing lipid peroxidation. (**D**) Acetylcholine (ACh), a neurotransmitter essential for processing memory and learning, is decreased in both concentration and function in AD. Decreased levels of ACh can be restored by anticholinesterase activity of various phytosterols. (**E**) ROS irreversibly oxidize DNA and are important mediators of Aβ-induced neuronal cell death in the development of AD. Abbreviations: APP: Amyloid Precursor Protein; AChE: Acetyl Cholinesterase Enzyme; BACE 1: Beta-Secretase 1; BChE: Butyl Cholinesterase Enzyme; Bcl-2: B-Cell Lymphoma 2; CAT: Catalase; GPX: Glutathione Peroxidase; HO 1: Heme Oxygenase; ROS: Reactive Oxygen Species; SOD: Superoxide Dismutase.

This section highlights various biological activities in vitro or in vivo by the major phytosterols, such as β-sitosterol, stigmasterol, and campesterol, and other minor sterols brassicasterol, lanosterol, 24(*S*)-saringosterol, and the 4,4-dimethyl sterols. The structures of the selected phytosterols included in this review are shown in Figure 4, and a summary of their implicated neuroprotective activities is shown in Table 1 and Figure 5.

### 4.1. β-Sitosterol

β–Sitosterol is one of the main dietary phytosterols belonging to the class of organic compounds known as stigmastanes. These are sterol lipids with a structure based on the stigmastane skeleton, which consists of a cholestane moiety bearing an ethyl group at the C-24 position. In a comprehensive study, the effects of β-sitosterol in behavioral studies were observed in transgenic mice using a shallow water maze (SWM), Y-maze, and balance beam tests. The in vivo (in frontal cortex and hippocampus) and in vitro anti-acetyl choline esterase (AChE), anti-butyl choline esterase (BChE) inhibitory potentials, antioxidant activity, and molecular docking were also analyzed [45,46]. β-sitosterol revealed strong Ache properties under both in vitro and in vivo conditions. An IC_50_ value of 55 and 50 μg/mL against AChE and BChE, respectively [45], and 62 μg/mL against both AChE and BChE [46] were reported in vitro, while the activity was significantly lower in brain tissue homogenates. The AChE inhibition was further confirmed by in silico studies in which β-sitosterol was strongly bound to the active sites of AChE and BChE through the *para*-hydroxyl group of the phenolic moiety networked with the active site water molecule and the side chain carbonyl residues through H-bonding. The rest of the active compound was packed in a shallow pocket through H-bonding [46]. In the antioxidant assays, the IC_50_ values were observed as 140, 120, and 280 μg/mL in the 2,2-diphenyl-1-picryl-hydrazyl-hydrate (DPPH), 2,2 -azino-bis (3-ethylbenzothiazoline-6-sulfonic acid) (ABTS), and hydrogen peroxide (H_2_O_2_) assays, respectively. The treatment group had lower oxidative stress in comparison to the disease control. β-Sitosterol was reported to increase levels of antioxidant enzymes by activating the estrogen receptor/PI3-kinase pathway. It also regulated glutathione levels, suggesting its role as an effective free radical scavenger [47,48]. Furthermore, glucose oxidase-mediated oxidative stress and lipid peroxidation might be inhibited from the incorporation of β-sitosterol into cell membrane, which showed valuable effects of this compound in neurodegenerative disorders including AD [47]. β-sitosterol also restored behavioral deficits, working memory, and motor coordination in transgenic animals [45]. It also hastened neuron degeneration in mice deficient for LXRβ [49], suggesting that LXRβ activation by non-glucosylated sitosterol is neuroprotective.

In another study, the effect and mechanism of β-sitosterol on deficits in learning and memory in amyloid protein precursor/presenilin 1 (APP/PS1) double transgenic mice was investigated. APP/PS1 mice were treated with β-sitosterol for 4 weeks, from the age of 7 months. Brain Aβ metabolism was evaluated using ELISA and Western blotting. β-sitosterol treatment ameliorated spatial learning and recognition memory capacity, along with reduction in plaque deposition and renovation of the excitatory postsynaptic current frequency in the hippocampus [50]. Substitution of cholesterol by β-sitosterol promoted non-amyloidogenic AP processing by migrating it to non-lipid raft region [41]. However, contradictory results were obtained in another study [31], where β-sitosterol increased the secretion of amyloid by 115.2% ± 2.2%.

Reports have suggested the role of cholesterol in amyloid precursor protein (APP) processing [40,51,52,53]; thus, cholesterol may be considered as a target for developing drugs to treat AD. Phytosterols are structurally similar to cholesterol and have been widely used to reduce blood cholesterol [54]. It was found that β-sitosterol efficiently repressed the release of high cholesterol-driven platelet Aβ. In addition, β-sitosterol also prevented high cholesterol-induced increases in β- and γ-secretase activity [55].

Mitochondrial dysfunction has a critical role in neuronal degeneration [56]. Therefore, augmentation of mitochondrial function by increasing the mitochondrial membrane potential (ΔΨm) and mitochondrial adenosine triphosphate (ATP) may be valuable in treating AD. Previously, it was suggested that an enhancement in mitochondrial ATP levels may be beneficial for neurodegenerative diseases [57]. Integration of β-sitosterol into mitochondrial membrane augmented the mitochondrial function by endorsing inner mitochondrial membrane fluidity, thereby raising the ∆Ψm and ATP concentrations. Hence, β-sitosterol could be an effective dietary therapy for neurodegenerative diseases such as AD [58].

Continual neuroinflammation in neurodegenerative diseases damages the neurons due to the release of toxic factors such as nitric oxide. In a study, β-sitosterol displayed anti-inflammatory action in BV2 cells upon exposure to LPS by reducing the expression of pro-inflammatory markers, such as interleukin-6 (IL-6), inducible nitric oxide (iNOS), tumor necrosis factor-α (TNF-α), and cyclooxygenase-2 (COX-2). It also suppressed the phosphorylation and degradation of inhibitor of nuclear factor kappa B (IκB) and inhibited the phosphorylation of nuclear factor kappa B (NF-κB) and extracellular signal-regulated kinase (ERK), which regulated various cytokines in inflammatory pathway [59].

All of these studies confirmed the multi-target potential of β-sitosterol in management of memory deficit disorders such as AD.

### 4.2. Stigmasterol

Stigmasterol is a steroid derivative having a hydroxyl group in position C-3 of the steroid skeleton, unsaturated bonds in position C-5/C-6 of the B ring, and alkyl substituents in position C-22/C-23. Stigmasterol is shown to be involved in neuroprotection through a multi-target approach. Stigmasterol isolated from *Rhazya stricta* fruits displayed in vitro AChE inhibitory activity with an IC_50_ of 644 ± 11.75 µM [60]. It was also reported to reduce amyloid plaques by decreasing the β-secretase cleavage of APP [31]. Stigmasterol displayed neuroprotective activity against glutamate-induced toxicity by inhibition of reactive oxygen species (ROS) and Ca^2+^ production in in vitro studies [61]. To investigate the possible neuroprotective mechanisms of stigmasterol, an H_2_O_2_-induced oxidative stress model in SH-SY5Y neuroblastoma cells was established [62]. H_2_O_2_ exposure raised the levels of ROS significantly within the cells, inducing apoptosis. However, pre-incubation with stigmasterol prevented oxidative stress-induced cell death by reducing the level of ROS in the cells. It also upregulated catalase, forkhead box O (FoxO) 3a, and anti-apoptotic protein B-cell lymphoma 2 (Bcl-2) in the neurons. Additionally, it also increased the expression levels of sirtuin 1 (SIRT1) and decreased the levels of acetylated lysine [62]. Sirtuins are highly conserved NAD (+)-dependent enzymes that have positive effects in age-related neurodegenerative diseases. In vitro and in vivo studies revealed that the increased SIRT1 protein ameliorated AD-like symptoms by reducing the memory decline [63]. Similarly, reduction of Aβ aggregations was reported through activation of SIRT1 causing upregulation of APP metabolism by α-secretase [64]. Moreover, overexpression of SIRT1 protected SH-SY5Y cells from toxicity-induced cell death [65]. Using docking studies, the possible interaction between stigmasterol and the activator binding site of SIRT1 was also inspected. According to the binding analysis, SIRT1 interacted with stigmasterol and resveratrol; both compounds bind to Thr^209^ and Pro^212^ by van der Waals and hydrophobic interactions, respectively. Moreover, they also shared binding interactions with Phe^414^, Asp^292^, Gln^294^, and Ala^295^ [62]. Stigmasterol was also reported to inhibit several pro-inflammatory cytokines in IL-1β-treated cells without affecting IL-6 levels, suggesting its role in the IL-1β-induced NF-κB inflammatory pathway [66].

Transcriptomic analysis implicated the role of stigmasterol in upregulating genes involved in neuritogenesis (*Map2, Dcx, Reln)* and synaptogenesis (*Arc, Egr1, Nr4a1)*, thereby stimulating neuronal architecture in primary hippocampal neurons for processing memory and learning. Stigmasterol also decreased the expression of K^+^ transport genes to sustain neuronal excitability under adverse conditions [67]. Thus, the broad spectrum of action has made stigmasterol a potential therapeutic candidate for the prevention and treatment of brain disorders, especially AD. Mice fed with stigmasterol-enriched diets exhibited the reduction of Aβ generation by diminishing β-secretase activity, decreasing expression of all γ-secretase components, reducing cholesterol and presenilin distribution in lipid rafts involved in amyloidogenic APP cleavage, and by decreasing β-secretase 1 (BACE1) internalization to endosomal compartments [31].

### 4.3. Campesterol

Campesterol is characterized by the presence of a hydroxyl group at C-3 of the steroid skeleton and saturated bonds throughout the sterol structure, with the exception of the C-5/C-6 double bond in the B ring. Campesterol only marginally affected Aβ secretion in APP695-expressing SH-SY5Y cells, mainly due to increased γ-secretase activity. The β-secretase activity was also significantly increased in purified membranes of mouse brains (104.8% ± 1.4), whereas no effect was observed in SH-SY5Y wild type (wt) membranes. Both gene expression and protein levels of BACE1 were unchanged. However, campesterol significantly increased γ-secretase activity in the purified membranes of SH-SY5Y wt cells and mouse brains (115.5% ± 3.0 and 106.9%, respectively). Gene expression of all components of the γ-secretase complex remained unchanged in the presence of campesterol, reflected by unchanged PS1 protein level [31].

In a clinical trial of AD patients, sitosterol and campesterol levels in the brain were almost similar in comparison to the healthy control group (6.3 ± 0.8 ng/mg and 6.2 ± 0.8 ng/mg versus 5.0 ± 0.8 ng/mg and 5.0 ± 0.8 ng/mg, *p* < 0.05, in the assayed and control groups, respectively). However, the levels of 27-hydroxycholesterol were decreased due to oxidative damage, where the BBB was not disturbed [68].

### 4.4. Brassicasterol

Brassicasterol is a 3β-sterol, that is, (22*E*)-ergosta-5,22-diene substituted by a hydroxy group at position 3β. It is found in marine algae, fish, and rapeseed oil. In the early stages of AD, the functions of the BBB and choroid plexus are impaired, resulting in reduced concentrations of phytosterols in cerebrospinal fluid (CSF). Both sitosterol and brassicasterol were significantly reduced, but when corrected for CSF cholesterol concentrations, only brassicasterol was reduced significantly in CSF of AD patients. Brassicasterol also amended the predictive value, when added to the biomarkers pTau and Aβ-42. Therefore, brassicasterol may be a novel additional CSF biomarker of AD patients [69]. Although their study was not designed to determine whether brassicasterol levels in CSF may be prognostic of AD progression, their observations nonetheless generated interesting hypotheses about the utility of plant sterol metabolism as a potential biomarker in AD.

However, in another study [31], brassicasterol directly increased β-secretase activity in purified membranes of SH-SY5Y wt cells and mouse brains (108.5% ± 1.9 and 106.6% ± 1.7, respectively) without affecting gene expression and protein level of BACE1. Similarly, γ-secretase activity was also slightly, but significantly, increased in purified membranes of SH-SY5Y wt cells and mouse brains (108.2% ± 1.0 and 111.4% ± 1.6%, respectively). Considerable changes in gene expression were only found for the γ-secretase components presenilin 2(PS2)/anterior-pharynx-defective protein 1 (APH1B), but not for the homologues PS1 and APH1A, presenilin enhancer 2 (PEN2) [31].

### 4.5. Lanosterol

Lanosterol is a Δ^8,24^ phytosterol formed from the cyclization of 2,3-oxidosqualene catalyzed by lanosterol synthetase [22]. It was shown to be a potential candidate in the treatment of cataracts by disrupting the aggregations of intracrystalline proteins [70]. Several studies suggested it as a promising modulator of neurodegenerative diseases [71,72,73,74]. In silico studies revealed that lanosterol disrupted the aggregations of the amyloidogenic KLVFFA peptide chain in Aβ_42_ by hydrophobic interaction with Phe-19 and Phe-20 and interfering with the β-sheet-β-sheet packing interactions. This was further correlated with the thioflavin T (ThT) fluorescence assay and atomic force microscope (AFM) imaging. Furthermore, lanosterol also revealed neuroprotective effects with PC12 cells upon Aβ_42_ treatment [71].

The aggregation of misfolded proteins in neuronal cells is interconnected with neurodegeneration. In vitro report utilizing HeLa and HEK-293A cells revealed that lanosterol and lanosterol synthase facilitated the renewal of the cell’s trapped misfolded proteins in intracellular proteostasis. Lanosterol effectively minimized the number and size of sequestosomes/aggresomes in HeLa and HEK-293A cells. These results suggested a cytoprotective property of lanosterol in facilitating cell proliferation and survival by disaggregating and refolding ubiquitinated misfolded proteins [72]. Lanosterol treatment reduced the accumulations and cytotoxicity of misfolded protein aggregations through induction of co-chaperone (CHIP) and by promoting autophagy [73].

The neuroprotective effects of lanosterol was also demonstrated in vivo [74]. Administration of lanosterol to 1-methyl-4-phenyl-1,2,3,6-tetrahydropyridine-treated mice showed lanosterol reduction in the nigrostriatal region, suggesting an altered lanosterol metabolism involvement in Parkinson’s disease pathogenesis. Treatment with lanosterol also indicated the increased cell viability in dopaminergic neurons, when cells were treated with 1-methyl-4-phenylridium, by regulating and inducing mitochondrial function and depolarization, and promoting autophagy.

### 4.6. 24(S)-Saringosterol

24(*S*)-Saringosterol or (3β)-stigmasta-5,28-diene-3,24-diol is a marine phytosterol commonly isolated from the edible seaweed *Sargassum fusiforme*. 24(*S*)-saringosterol was shown to be a potential anti-atherosclerotic natural agent by lowering cholesterol and a selective LXR-β agonist [75,76]. An in vivo study of APPswePS1DE9 mice treated with 24(*S*)-saringosterol for 10 weeks revealed a slowing of cognitive decline based on enhanced spatial and object memory assessments. It also reduced the in vivo expression of ionized calcium-binding adapter molecule 1 (Iba1), a marker for microglial activation and inflammation. No effect was detected on the amount of Aβ plaques, hence suggesting the neuroinflammatory effects of 24(*S*)-saringosterol in the deterrence of cognitive decline or limited penetration through BBB [77].

Dietary supplementation of *S. fusiforme* enriched in 24(*S*)-saringosterol in an AD mouse model indicated a reduction in hippocampal Aβ plaques and improvement in short-term memory. In vitro treatment with the same extract on mouse neuroblastoma (N2a) cells also exhibited a reduced secretion of Aβ plaques [78]. The reduction in the levels of Aβ plaques by dietary supplementation of *S. fusiforme* extracts enriched with phytosterols including 24(*S*)-saringosterol may indicate a synergistic model of the phytosterols in the prevention of neurodegenerative diseases.

### 4.7. 4,4-Dimethyl Phytosterols

4,4-Dimethyl phytosterols are a class of bioactive compounds having two methyl groups at the C-4 position of the aliphatic A-ring. Significant amounts of α-amyrin, β-amyrin, taraxerol, and lupeol are present in plants and vegetable oils. In scopolamine-induced cognitive impairment in mice, elevated levels of memory-related proteins in hippocampus were reported in the presence of α-amyrin and β-amyrin. Improvement in cognitive function was found to be induced through the activation of extracellular signal-regulated kinase (ERK) and glycogen synthase kinase-3β (GSK-3β). Additionally, β-amyrin and not α-amyrin displayed anti-AChE activity [79,80]. In scopolamine- and streptozotocin-induced memory deficit studies in mice, taraxerol also displayed anti-AChE activity through activation of the AChE receptor system [81]. Molecular docking studies confirmed high affinity of taraxerol for fibrils and Aβ [82]. Lupeol was found to inhibit BACE1 in both enzymatic and docking studies and is therefore a promising candidate for AD treatment [83,84]. The reduced levels of LDL-C were typically related to the prevalence of AD and PD [85]. Consequently, the anti-Alzheimer’s and anti-Parkinsonian potential of 4,4-dimethy sterols may also be linked to their cholesterol metabolism regulating activity [86].

In addition, the elimination of the misfolded proteins through autophagy is an important mechanism in preventing neurodegenerative diseases. It was found that β-amyrin participated in the LGG-1 (ubiquitin-like modifier involved in the formation of autophagosomes) related autophagy pathway by improving LGG-1 expression and exhibiting a protective effect on dopaminergic neurons by decreasing cell damage, and α-synuclein aggregation, which improves PD symptoms [87].

### 4.8. Ergosterol

Ergosterol is the most abundant fungal phytosterol bearing the Δ^5,7^ diene oxysterol skeleton [88]. In in vitro β- and γ-secretase assays utilizing N2a cells, ergosterol slightly decreased the β-secretase activity at 20–80 µM concentrations, while strongly inhibiting the γ-secretase activity at 40 µM [53].

These data suggest the promising potentials of the phytosterols as modulators of neurodegenerative diseases. The neuroprotective effects exhibited by the phytosterols are summarized in Table 1.

**Table 1:** Neuroprotective mechanisms of the reported phytosterols.

Phytosterol	Mode of Action	Study	References
β–Sitosterol	AChE and BChE inhibitory activity	in vivo (mice), in vitro, in silico	[45,46]
	Increased levels of antioxidant enzymes by activating estrogen receptor/PI3-kinase pathway	in vitro (RAW 264.7; HT22)	[47,48]
	Anti-inflammatory	in vitro (BV12)	[59]
	Increase mitochondrial potential	in vitro (HT22)	[58]
Stigmasterol	AChE inhibitory activity	in vitro	[60]
	Reduced the β-secretase activity. Reduced the expression of all γ-secretase components Reduced the cholesterol and presenilin distribution in lipid rafts implicated in amyloidogenic APP cleavage. Decreased the BACE1 internalization to endosomal compartments, involved in APP β-secretase cleavage	in vivo (mice), in vitro (HT22)	[31,55]
	Decrease ROS	in vitro (SH-SY5Y)	[61,62]
	Anti-inflammatory	in vitro (mouse chondrocytes and human osteoarthritis chondrocytes)	[66]
Brassicasterol	Marker in CSF of AD patients	cerebrospinal fluid (CSF)	[69]
	Minor or no effect on Aβ secretion	in vivo (mice)	[31]
Campesterol	Minor or no effect on Aβ secretion	in vivo (mice)	[31]
Lanosterol	Reduced the accumulations and cytotoxicity of Aβ aggregation through induction of co-chaperone and by promoting autophagy	in silico, in vitro (HeLa, PC12, HEK-293A), in vivo (mice)	[71,72,73,74]
24(*S*)-Saringosterol	Reduced the in vivo expressions of Iba1	in vivo (mice)	[77]
Dietary supplementation of *S. fusiforme* enriched in 24(*S*)-saringosterol	Reduced secretion of Aβ plaques. Improves memory in AD mice model	in vivo (mice), in vitro (N2a)	[78]
α-Amyrin	Elevated levels of memory related proteins through the activation of ERKGSK-3β	in vivo (mice)	[79]
β-Amyrin	Elevated levels of memory related proteins through the activation of ERKGSK-3β. AChE inhibitory activity	in vivo (mice)	[79,80]
Taraxerol	AChE inhibitory activity	in vivo (mice)	[83]
	High affinity of taraxerol for fibrils and amyloid- β	in silico	[84]
Lupeol	BACE-I inhibitory activity	in vitro, in silico	[85,86]
Ergosterol	Reduced the β- and γ-secretase activity	in vitro	[44]

## 5. Physiological versus Pathological Features of Phytosterols

In diet, phytosterols primarily reduce blood cholesterol concentrations [89] by lowering serum LDL-cholesterol concentrations, with no effect on either serum HDL-cholesterol concentrations or triacylglycerol levels [90]. They are also anti-inflammatory and have physiological functions such as growth regulation and the promotion of protein synthesis, immune regulation, and hormone-like effects [91]. The hypocholesterolemic effect of 4-desmethyl sterols is well known [92], whereas 4,4-dimethylsterols (lupeol, α-amyrin, cycloartenol) have limited action on cholesterol reduction [93]. As phytosterols reduce the solubility of cholesterol, some other lipophilic compounds such as lipophilic antioxidant nutrients may also be displaced. Randomized trials have shown that phytosterols lower the blood concentrations of β-carotene (by about 25%), α-carotene (by 10%), and vitamin E (by 8%) [94,95], which protect the oxidation of LDL cholesterol. However, after the action of phytosterols, β-carotene was found to be reduced by 8–19%, while fat-soluble vitamins (A, D, E, K) remained unchanged [94]. Therefore, intake of food rich in carotenes may balance this side effect.

The most adversarial effect of phytosterols is seen in a rare inherited disease, sitosterolemia, described by tendon xanthoma and premature coronary disease [96]. These patients have mutated ABCG5 and ABCG8 transporters that cause reduced transport of phytosterols from enterocytes back into the intestinal lumen and reduced secretion into bile. Hence, these patients have increased phytosterol absorption (3–4 fold) and low biliary excretion, resulting in build-up of these compounds in plasma and tissues [97]. A relationship between increased plasma phytosterols (7–16%) and increased risk of coronary heart disease (CHD) has been reported [98]. However, various contradictory reports rule out any relation between phytosterols and the risk of incident CHD. Additionally, plasma concentrations of the main phytosterols (sitosterol and campesterol) can be used as bio markers for cardiometabolic risk, as moderately elevated plasma sitosterol, but not campesterol, may possibly indicate decreased risk for CHD [14]. Thus, the available data are contradictory and more in-depth studies are required to confirm whether phytosterols are friend or foe.

## 6. Safety Concerns for the Phytosterols

Even though intake of phytosterols (up to 3 g/day) is considered safe by the FDA, information on their toxicity is limited, including their indistinct anabolic effect [18]. No adverse effect of phytosterols on mental or cognitive activity has been reported yet. However, oxidized phytosterols were reported to exhibit neurotoxicity with glutamate excitotoxicity and ROS generation [87,99]. Several unfavorable effects of phytosterols on endothelium-dependent vasorelaxation in *wt* mice have been reported [100,101,102,103]. The types and concentrations of phytosterols or their structure may determine and influence the production of superoxides by endothelial cells [101,102,103]. Collectively, these studies indicated that phytosterols could modulate various endothelium-dependent processes, such as vasorelaxation, oxidative stress, ischemia–reperfusion, and neuroinflammation, which are key biological processes in the progression of CNS disorders. Therefore, depending on the nature, concentrations, and target cells, the phytosterols may have critical influences on neurodegenerative disorders [104].

## 7. Future Perspectives and Conclusions

Phytosterols are an important component of diet. They have wide applications in foods and cosmetics. To date, our knowledge has been limited to their cholesterol-lowering properties, protective effects mostly against cardiovascular diseases, and their anti-cancer and anti-inflammatory potential. Extensive research on the use of phytosterols for the preventive and therapeutic management of other diseases should be highly explored. The impact of phytosterols on the central nervous system is another exciting avenue of investigation. Phytosterols could cross the BBB through a less-known mechanism and become accumulated in the brain. The BBB is one of the most important and largest barriers among the three CNS barriers for the exchange of constituents between the blood and the CNS [105]. Any impairment in the brain endothelial cells may alter the normal function of adhesion molecules, chemotactic proteins, and angiogenic factors; thus, increased ROS production, infiltration of immune cells, and neuroinflammation could be the consequences in CNS disorders [106,107,108,109]. Moreover, normal functioning of various signaling pathways occurring at lipid rafts (cholesterol-rich domains), such as γ-aminobutyric acid and glutamate signaling, may perturb the synaptic vesicle turnover, protection of motor neurons by brain-derived neurotrophic factor (BDNF), functioning of the calcium channel, and calcium-dependent neurotransmitter release [110,111,112,113].

AD patients are shown to have compromised cholesterol turnover [114,115], where suppressed cholesterol biosynthesis may reduce the production of Aβ both in vitro and in vivo [68]. Likewise, a cholesterol-rich diet increased the generation of Aβ in mice [116,117,118]. The increased cellular cholesterol turnovers were in association with increased expression of the LXR-activated genes, which significantly amended cognitive performance in AD animal models [119,120,121].

Because the early stage of AD is linked to BBB dysfunction, the decreased concentrations of phytosterols in CSF could be used as a promising prognostic biomarker. Importantly, cholesterol is highly amyloidogenic, whereas the phytosterols are not. In fact, phytosterols significantly reduced brain Aβ levels and β- and γ-secretase activities in vivo, suggesting that a diet enriched in plant sterols might be beneficial for neurodegenerative diseases. Research on the optimal doses of phytosterols through oral administration would be essential. Because the consumed phytosterols would have limited intestinal absorption and crossing the BBB may take longer periods of ~6 weeks, higher doses may be required to capture the benefits [122]. Researchers have resorted to nanoencapsulation for improving the bioavailability of phytosterols in the food industry [123,124,125], but only for research purposes as a methodology of drug delivery. Thus, future studies should be carried out to explore the therapeutic and disease-specific mechanisms of phytosterols for their neuroprotective role in neurodegenerative diseases.

## Data Availability

Not applicable.
